# Early-onset psychosis in an adolescent with DiGeorge syndrome: A case report

**DOI:** 10.4102/sajpsychiatry.v24.i0.1164

**Published:** 2018-02-21

**Authors:** Keneilwe Molebatsi, Anthony A. Olashore

**Affiliations:** 1 Department of Psychiatry, University of Botswana, Botswana

## Abstract

DiGeorge syndrome (DGS) was first described in 1829 by Dr Angelo DiGeorge. DGS is a cluster of symptoms because of a defect in the development of the pharyngeal pouch. Evidence from cytogenetic studies has linked the pathogenesis of DGS with a deletion of a gene located in chromosome 22-band 22q11. In most affected individuals, the deletion is *de novo*; however, inheritance has been reported in 10% – 25% of patients. DGS commonly presents with a classical triad of conotruncal cardiac anomalies, hypoplastic thymus and hypocalcaemia. DGS may be of focus to a psychiatrist as it is associated with cognitive deficits, high rates of schizophrenia and anxiety disorders. Patients may also present to mental health care workers with learning disabilities, developmental delay and behavioural disorders such as attention-deficit or hyperactivity disorder. Mental health workers therefore play an invaluable role in the diagnosis and timely treatment of the disorder. In a resource-limited area such as Botswana, with scarce mental health professionals, paediatricians and neurologists, DGS may be frequently misdiagnosed with consequent inappropriate interventions that may increase morbidity. Herein, we present a case to raise awareness and demonstrate one of the varied ways the syndrome may present. The multifaceted nature of DGS presentation underscores the need for a multidisciplinary approach to treatment.

## Background

Angelo DiGeorge first described DiGeorge syndrome (DGS) in 1829 as a congenital absence of the thymus and parathyroid glands.^1^ The discovery that patients with DGS had a deletion of a gene in chromosome 22q11.2 occurred in the 1980s.^2^

DiGeorge syndrome is the second most common genetic cause of developmental delay after Down syndrome; however, it remains clinically unrecognised because of its varied presentations.^3^ It has an estimated prevalence of 1 in 4000 live births, with males and females being equally affected.^4^ One should have a high index of suspicion in patients who present with two or more of the following: immunodeficiency, developmental delays, learning disabilities, behavioural disorders, other psychiatric symptoms, cardiac anomalies and hypocalcaemia.^4,5^

Research has demonstrated a strong association between DGS and psychiatric disorders such as psychotic disorders, attention-deficit or hyperactivity disorder, mood disorders, anxiety disorders and autistic spectrum disorders (ASD).^3,5^ Of the behavioural phenotype of DGS, elevated risk of psychosis, particularly schizophrenia, is the most disturbing feature, accounting for a 30-fold increase compared to the general population.^3^

A case report of a patient with chromosome 22q11.2 deletion and later diagnosed with schizophrenia is provided.

## Case report

A 13-year-old female Motswana student, who is the first born in a family of two children and being raised by a single unemployed mother, was referred to a psychiatric hospital by her local facility. She presented with a 1 week history of calling out for people who were not there as if she were conversing with them and seeing things other people could not see. She was also reported to often appear anxious and was not sleeping well at night. The symptoms appeared to worsen daily, prompting the caregivers to seek help. There have been no preceding life events that may have precipitated the symptoms, and she had never been admitted or been on treatment for any psychiatric disorder before the current presentation.

Ms K was born via a normal vaginal delivery at 32 weeks gestational age, with a birth weight of 2.1 kg. The mother was Gravida 2, Para 2, and antenatal history was unremarkable. Her mother highlighted that the patient had delayed developmental milestones as she did not walk and talk until after age two. Regarding social development, Ms K was reported to prefer solitary activities, and if she interacted with others, she would choose children younger than her. She reportedly attended a normal stream school for 2 years but was transferred to a special needs school (i.e. school for children with learning difficulties) because of academic difficulties.

On medical history, she has been diagnosed with a complex congenital heart disease: pulmonary atresia, large ventricular septal defect, pulmonary regurgitation, right ventricular hypertension and failure. She underwent corrective surgeries for the cardiac conditions at ages 6 years and 12 years, an umbilical herniorrhaphy at 3 years and clubfoot repair at 2 years.

Typical features of DGS on physical appearance were a broad flat nose, small ears and a thoracolumbar scoliosis, whereas the typical long face, hypertelorism and micrognathia were absent. Physical examination revealed a mediastinal scar and a pansystolic murmur. Her blood pressure was 113/83 mmHg, pulse rate was 114 beats/minute and temperature was 35.8 °C. Investigations such as full blood count, liver function test, urea and electrolytes were within normal ranges. A chromosomal analysis was positive for chromosome 22 deletion syndrome.

Mental status assessment on the index consultation revealed a well-nourished adolescent. She was very restless, pacing up and down the interview room, thus making it difficult to establish a rapport. There was no eye contact. She had a labile affect. She was socially inappropriate as she kept undressing during the interview. Thought process was mostly tangential. She had fixed belief that her family members were bewitching her. She reported that God was commanding her to take her clothes off and she admitted to seeing a short man in the interview room.

A working diagnosis of acute schizophrenia-like psychotic disorder was made using the International Classification of Diseases-10 (ICD-10) diagnostic criteria. The patient was admitted on haloperidol 3 mg at night. Four days post admission, she was noted to have increased motor activity, restlessness and sialorrhea, whereas the psychotic symptoms persisted. Manic symptoms such as elated or irritable mood and increased energy were absent. On suspicion of akathisia, haloperidol was stopped and she was started on olanzapine, 5 mg once daily at night. The extra pyramidal side effects (EPSE) symptoms reduced after 2 days. Psychotic symptoms subsided on day nine post admission. She stabilised 2 weeks after admission and was discharged on olanzapine 5 mg once daily at night. She was reviewed in an outpatient clinic after 2 weeks and remained stable. She continued reviews as an outpatient for 4 months and medication dose was reduced to 2.5 mg nocte. A month later, she presented to OPD with history of poor sleep at night, laughing inappropriately and isolating self. Medication was reviewed upwards to olanzapine 5 mg nocte as it was on discharge. For long-term management, patient was to be enrolled in vocational training and behavioural therapy. A definitive diagnosis of early-onset schizophrenia in a patient with DiGeorge syndrome was made.

## Ethical consideration

Written informed consent for the publication was obtained from the patient’s mother.

## Discussion

The index case typifies the early-onset schizophrenia mostly observed in children with DGS. In contrast to other commonly co-occurring psychiatric disorders mentioned previously, the elevated risk for psychotic disorders such as schizophrenia suggests that it is more specific to DGS rather than a consequence of developmental delays.^6^

Research findings have shown that low levels of proline dehydrogenase (PRODH) and catechol O-methyltransferase (COMT) in DGS are responsible for the phenotypic representation of psychotic disorders.^6^ Whilst the COMT gene expression produces the COMT enzyme which metabolises catecholamines, the PRODH encodes a mitochondrial enzyme that degrades the amino acid proline.^6^ Although this pathophysiology is shared with ASD, symptoms suggestive of ASD in patients with DGS are unlikely to indicate a prodrome of schizophrenia.^6^ It has also been suggested that haploinsufficiency for specific genes that are necessary for neurodevelopment leads to reduced synaptic plasticity and formation of aberrant connectivity, thus setting the stage for increased vulnerability to psychosis in individuals with DGS.^7^

Some authors have argued that with respect to the core clinical features, schizophrenia without DGS is largely indistinguishable from ‘DGS-schizophrenia’.^8^ Nonetheless, some differences in ancillary clinical features such as lower rate of comorbid substance-use disorders have been noted.^8,9^ In addition, children with DGS-schizophrenia could have neurobehavioral physiognomies other than schizophrenia’s central symptoms.^9^ For example, greater severity of excitement and impulsivity which may be associated with short-lived irritability or emotional outbursts are commonly observed in patients with ‘DGS-schizophrenia’.^9,10^ This may explain the emotional lability and restlessness observed in our patient on admission. Unlike in manic episodes where there are more prolonged mood changes, these features were short lived without any exacerbation as reported by earlier authors and were dominated by psychotic symptoms.^11^

Prior to the index episode, the patient’s mother observed some odd and unusual behaviours such as self-absorption, solitude, anxiety and preference for much younger children. Earlier literature have suggested the possibility of picking up some predictive symptoms prior to the development of psychosis.^5,12^ For example, subthreshold psychotic symptoms such as odd or eccentric symptoms as observed in our index patient are often reported by parents of children with DGS in 30% – 50% of cases.^13^ Another useful predictor of psychosis in children with DGS is lower verbal IQ, as an average of 10-point decline in verbal IQ from baseline has been linked to later development of psychotic disorders among the patients.^5,12^ Albeit no structured IQ test was performed because of lack of necessary expertise, her inability to cope in a regular school (i.e. attending a learning disabled school), delayed speech and communication are suggestive of below average IQ.

In most cases, these predictive symptoms of mental disorders are missed, because patients with DGS are more likely to present first to non-mental health experts. With the exception of one previously reported case, it is uncommon for individuals with DGS to present with behavioural symptoms before the physical manifestations.^14^ The present case is not an exception, as she typically presented first with physical phenotypes typical of DGS, which include cardiac anomaly; skeletal deformities, such as clubbed foot and scoliosis; and facial features, for example, flat nose and small ears.^2,4^ With these medical conditions, psychiatrists are usually not involved until much later, when clear manifestations of mental disorders are observed. It is therefore not surprising that the index episode of our patient is her first contact with psychiatrists, despite previous history of odd behaviours and symptoms of low IQ. The benefits of better outcomes that are associated with early treatment of psychiatric disorders underscore the need for attending physicians to purposefully screen for these predictors and involve the psychiatrists as appropriate.

Treatment of psychosis in children with DGS is essentially the same as in the general population.^5^ Antipsychotics, preferably the newer agents such as risperidone, olanzapine and quetiapine, are generally recommended and better tolerated by children with DGS.^15^ It is also advocated that antipsychotic use in children with DGS should ‘start low and go slow’, to prevent EPSE and other metabolic side effects.^5,15^ In this case, symptoms of EPSE were observed with the use of older-generation antipsychotic medication, and these subsided when it was replaced with a minimum effective dose of olanzapine (a newer agent). Notwithstanding the fact that no clinical guidelines concerning the management of ‘DGS-schizophrenia’ exist in Botswana, the above recommendations were followed in the management of our patient.

## Conclusion and recommendation

This case reiterates the need for paediatricians or other attending physicians to be aware of the behavioural manifestations of DGS, such as schizophrenia, while being wary of physical comorbidities that may complicate treatment outcomes. Therefore, it is recommended that attending physicians should as a rule screen for the predictors of psychosis and other behavioural phenotypes of DGS and involve the psychiatrists as appropriate. This will afford these individuals early opportunities for treatment and better outcomes.

## References

[1] Lischner HW, Dacon C, DiGeorge AM Normal lymphocyte transfer (NLT) test: Negative response in a patient with congenital absence of the thymus. Transplantation. 1967;5(3):555–556. 10.1097/00007890-196705000-00020 6032922

[2] de la Chapelle A, Herva R, Koivisto M, Aula P A deletion in chromosome 22 can cause DiGeorge syndrome. Hum Genet. 1981;57(3):253–256. 10.1007/BF00278938 7250965

[3] Jonas RK, Montojo CA, Bearden CE The 22q11.2 deletion syndrome as a window into complex neuropsychiatric disorders over the lifespan. Biol Psychiatry [serial online]. 2014 [cited 2017 Nov 10];75(5):351–360. Available from: http://www.ncbi.nlm.nih.gov/pubmed/23992925 10.1016/j.biopsych.2013.07.019 PMC3875621 23992925

[4] De Decker HP, Lawrenson JB The 22q11.2 deletion: From diversity to a single gene theory. Genet Med [serial online]. 2001 [cited 2017 Nov 10];3(1):2–5. Available from: http://www.ncbi.nlm.nih.gov/pubmed/11339372 10.1097/00125817-200101000-00002 11339372

[5] Tang KL, Antshel KM, Fremont WP, Kates WR Behavioral and psychiatric phenotypes in 22q11.2 deletion syndrome. J Dev Behav Pediatr [serial online]. 2015 [cited 2017 Nov 10];36(8):639–650. Available from: http://www.ncbi.nlm.nih.gov/pubmed/26372046 10.1097/DBP.0000000000000210 PMC4586411 26372046

[6] Radoeva PD, Coman IL, Salazar CA, et al Association between autism spectrum disorder in individuals with velocardiofacial (22q11.2 deletion) syndrome and PRODH and COMT genotypes. Psychiatr Genet [serial online]. 2014 [cited 2017 Nov 10];24(6):269–272. Available from: http://www.ncbi.nlm.nih.gov/pubmed/25325218 10.1097/YPG.0000000000000062 PMC4284058 25325218

[7] Prasad SE, Howley S, Murphy KC Candidate genes and the behavioral phenotype in 22q11.2 deletion syndrome. Dev Disabil Res Rev [serial online]. 2008 [cited 2017 Nov 10];14(1):26–34. Available from: http://www.ncbi.nlm.nih.gov/pubmed/18636634 10.1002/ddrr.5 18636634

[8] Duijff SN, Klaassen PWJ, de Veye HFNS, Beemer FA, Sinnema G, Vorstman JAS Cognitive development in children with 22q11.2 deletion syndrome. Br J Psychiatry [serial online]. 2012 [cited 2017 Nov 10];200(6):462–468. Available from: http://www.ncbi.nlm.nih.gov/pubmed/22661678 10.1192/bjp.bp.111.097139 22661678

[9] Bassett AS, Chow EWC, AbdelMalik P, Gheorghiu M, Husted J, Weksberg R The schizophrenia phenotype in 22q11 deletion syndrome. Am J Psychiatry [serial online]. 2003 [cited 2017 Nov 10];160(9):1580–1586. Available from: http://www.ncbi.nlm.nih.gov/pubmed/12944331 10.1176/appi.ajp.160.9.1580 PMC3276594 12944331

[10] Gothelf D, Frisch A, Munitz H, et al Clinical characteristics of schizophrenia associated with velo-cardio-facial syndrome. Schizophr Res [serial online]. 1999 [cited 2017 Nov 10];35(2):105–112. Available from: http://www.ncbi.nlm.nih.gov/pubmed/9988847 10.1016/s0920-9964(98)00114-5 9988847

[11] Aneja A, Fremont WP, Antshel KM, et al Manic symptoms and behavioral dysregulation in youth with velocardiofacial syndrome (22q11.2 deletion syndrome). J Child Adolesc Psychopharmacol. 2007;17(1):105–114. 10.1089/cap.2006.0023 17343558

[12] Antshel KM, Shprintzen R, Fremont W, Higgins AM, Faraone SV, Kates WR Cognitive and psychiatric predictors to psychosis in velocardiofacial syndrome. J Am Acad Child Adolesc Psychiatry [serial online]. 2010 [cited 2017 Nov 10];49(4):333–344. Available from: http://www.ncbi.nlm.nih.gov/pubmed/20410726 PMC2918883 20410726

[13] Baker KD, Skuse DH Adolescents and young adults with 22q11 deletion syndrome: Psychopathology in an at-risk group. Br J Psychiatry [serial online]. 2005 [cited 2017 Nov 10];186(2):115–120. Available from: http://www.ncbi.nlm.nih.gov/pubmed/15684233 10.1192/bjp.186.2.115 15684233

[14] Kook SD, An SK, Kim KR, Kim WJ, Lee E, Namkoong K Psychotic features as the first manifestation of 22q11.2 deletion syndrome. Psychiatry Investig. 2010; 7(1):72. 10.4306/pi.2010.7.1.72 PMC2848773 20396437

[15] Dori N, Green T, Weizman A, Gothelf D The effectiveness and safety of antipsychotic and antidepressant medications in individuals with 22q11.2 deletion syndrome. J Child Adolesc Psychopharmacol. 2017; 27(1):83–90. 10.1089/cap.2014.0075 26131914

